# A HORMONAL INFLUENCE? Polycystic ovary syndrome and borderline personality disorder

**DOI:** 10.1192/j.eurpsy.2024.375

**Published:** 2024-08-27

**Authors:** S. Jesus Magueta, M. Almeida, E. Osório, C. Guerra

**Affiliations:** Departamento de Psiquiatria e Saúde Mental, Centro Hospitalar do Baixo Vouga, Aveiro; ^2^Serviço de Psiquiatria, Hospital de São João, Porto, Portugal

## Abstract

**Introduction:**

Borderline Personality Disorder (BPD) is a chronic personality disorder characterized by emotional and interpersonal instability, difficulty in mentalization, impulsivity with functional impairment and increased rates of comorbid mental disorders. Polycystic ovary syndrome (PCOS) is the most prevalent endocrine disorder in premenopausal women, with important impact on quality of life and mental health. Studies have begun to explore the eventual relationship between these two pathologies.

**Objectives:**

The authors aim to describe the existing evidence exploring the relationship between BPD and PCOS as well as explore eventual common causal pathways and the forms which one might influence the other.

**Methods:**

The authors describe a clinical case of a 31 year old female patient with history of borderline personality disorder and polycystic ovary syndrome presenting with hyperandrogenism and hirsutism as well as menstrual irregularities. As a compliment to the case, the authors conducted a brief non-structured literature review using articles published in the Medline/Pubmed, ScienceDirect and Google Scholar databases. The keywords used during the research, alone or in combination, included: Polycystic ovary syndrome and Borderline Personality Disorder. The studies consulted in this work included: cross-sectional studies, cohort studies, literature reviews and clinical case reports. Of these, those that were written in the English language and deemed most pertinent to the explored theme were chosen for review in this work.

**Results:**

The results demonstrate a paucity in the literature with only 10 articles having been published between 2009 and 2023 having dedicated studies and research to the relationship between the pathologies. One study reports that those with PCOS show relevant psychiatric disorders in comparison to controls, including personality disorders, such as is demonstrated in the described clinical case. Of the few case studies available, these found that BPD was associated with PCOS with the latter having most frequently been diagnosed previously to the personality disorder. Altered androgen metabolism has been described in both these pathologies, thus further strengthening the relationship between these.

**Conclusions:**

Hormonal fluctuation has been classically associated with psychopathological symptoms, including unstable mood and impulsivity. The alterations demonstrated in PCOS might serve as an exacerbating factor in the genesis of the emotional instability and other symptoms present in BPD. The literature on this topic is still in an embryonic phase with a clear lacuna existing which merits attention and further study so as to fully comprehend the potential of these comorbid states. Clinicians should remain attentive to this comorbidity and the influence that PCOS might have on the psychopathology of BPD so as to better quality of life and global functioning which is impacted in both.

**Disclosure of Interest:**

None Declared

